# Lung Cancer Staging: Imaging and Potential Pitfalls

**DOI:** 10.3390/diagnostics13213359

**Published:** 2023-11-01

**Authors:** Lauren T. Erasmus, Taylor A. Strange, Rishi Agrawal, Chad D. Strange, Jitesh Ahuja, Girish S. Shroff, Mylene T. Truong

**Affiliations:** 1 Department of Anatomy and Cell Biology, Faculty of Sciences, McGill University, Montreal, QC H3A 0G4, Canada; lauren.erasmus@mail.mcgill.ca; 2 Department of Pathology, University of Texas Medical Branch, Galveston, TX 77555, USA; tastrang@utmb.edu; 3 Department of Thoracic Radiology, University of Texas MD Anderson Cancer Center, Houston, TX 77030, USA; ragrawal1@mdanderson.org (R.A.); cdstrange@mdanderson.org (C.D.S.); jahuja@mdanderson.org (J.A.);

**Keywords:** lung cancer, staging, TNM, CT, PET/CT

## Abstract

Lung cancer is the leading cause of cancer deaths in men and women in the United States. Accurate staging is needed to determine prognosis and devise effective treatment plans. The International Association for the Study of Lung Cancer (IASLC) has made multiple revisions to the tumor, node, metastasis (TNM) staging system used by the Union for International Cancer Control and the American Joint Committee on Cancer to stage lung cancer. The eighth edition of this staging system includes modifications to the T classification with cut points of 1 cm increments in tumor size, grouping of lung cancers associated with partial or complete lung atelectasis or pneumonitis, grouping of tumors with involvement of a main bronchus regardless of distance from the carina, and upstaging of diaphragmatic invasion to T4. The N classification describes the spread to regional lymph nodes and no changes were proposed for TNM-8. In the M classification, metastatic disease is divided into intra- versus extrathoracic metastasis, and single versus multiple metastases. In order to optimize patient outcomes, it is important to understand the nuances of the TNM staging system, the strengths and weaknesses of various imaging modalities used in lung cancer staging, and potential pitfalls in image interpretation.

## 1. Introduction

In 2020, lung cancer accounted for 1.8 million deaths worldwide [1,2]. Initial staging of the disease is typically performed with computed tomography (CT) and positron emission tomography/computed tomography (PET/CT). TNM-8 used a database of 94,708 patients from 1999 to 2010 collected from 35 centers in 16 countries [3]. This staging system is used for clinical and pathologic lung cancer staging for all histologic subtypes of the disease [3]. Since 2016, the TNM staging system has been established as the standard system of lung cancer staging by the Union for International Cancer Control (UICC) and the American Joint Committee on Cancer (AJCC). Histologic subtypes of lung cancer, such as non-small cell lung cancer (NSCLC), small cell lung cancer (SCLC), and bronchopulmonary carcinoid tumor, can all be clinically and pathologically staged using the TNM classification [4]. This staging system stratifies patient survival by using three factors to determine the anatomical extent of the malignancy: primary tumor (T), nodal metastasis (N), and metastasis to intra- and extrathoracic sites (M) (Table 1 and Table 2). For patients with small cell lung cancer, TNM-8 has been validated by an analysis of more than 5000 patients in the IASLC database [5]. For patients with bronchopulmonary carcinoid tumors, which account for 1–2% of all lung cancers with approximately 2000–4500 newly diagnosed cases each year in the United States, the combined stage categories provide useful information on outcomes for typical and atypical carcinoids [6]. However, due to persistent overlap in combined stage and subcategories of the staging system, the usefulness of the TNM staging system, particularly in the intermediate stages, is limited for carcinoid tumors [7].

Clinical staging is based on information obtained from physical examination, laboratory tests, imaging studies, and procedures (e.g., endobronchial ultrasound-guided nodal biopsy and mediastinoscopy but not from thoracotomy) performed to evaluate the scope of disease. Pathologic staging builds on clinical staging by adding information obtained upon surgical resection of the tumor, lymph nodes or metastases [8]. Accurate imaging interpretation is crucial for evaluating the extent of disease during clinical staging as it determines prognosis and allows for the development of a treatment plan tailored to the patient’s disease. Therefore, optimal patient care requires an understanding of the TNM staging system and the strengths and limitations of current imaging modalities used in lung cancer staging. In this article, we examine the role of computed tomography (CT) and fluorine-18fluoro-2-deoxy-D-glucose (FDG) positron emission tomography/computed tomography (PET/CT) in lung cancer staging using the TNM-8 system and review potential pitfalls in imaging interpretation.

## 2. T Classification

The T classification describes tumor size, degree of local invasion, and the presence and location of separate tumor lung nodules. IASLC recommends measuring the primary tumor to the nearest millimeter on contiguous 1 mm sections with lung window setting on any plane that exhibits the largest diameter. For solid and pure ground glass lung cancers, the longest diameter of the tumor is used for staging purposes [9,10,11]. For part-solid lung malignancies, the long axis diameter of the solid component should be recorded, as this is thought to represent the invasive component on pathology [9,12]. In practice, over three-quarters of radiologists in one investigation performed tumor length measurements on only the axial plane [11]. Except for a perfect sphere, axial plane only measurement is nearly always shorter than the longest diameter [11,13,14]. Numerous studies have demonstrated that by using only the axial plane to measure the longest dimension, the T category is underestimated by at least one level in 18–27% of cases [11,13,14].

Statistical analysis of the TNM-8 database showed significant differences in survival for each tumor size cut point. The size thresholds included the 3 cm cut point to separate T1 from T2 tumors, with a decline in survival associated with each 1 cm increment increase [15]. T1 tumors were categorized into three subgroups: T1a measuring 1 cm or less, T1b measuring more than 1 cm but equal to or less than 2 cm, and T1c measuring more than 2 cm but equal to or less than 3 cm [3]. Similarly, T2 malignancies were categorized into two subgroups with T2a tumors measuring more than 3 cm and less than or equal to 4 cm, and T2b lesions measuring more than 4 cm and less than or equal to 5 cm [3]. T3 lesions measure more than 5 cm and less than or equal to 7 cm, and T4 lesions account for malignancies greater than 7 cm.

**Figure 1:**
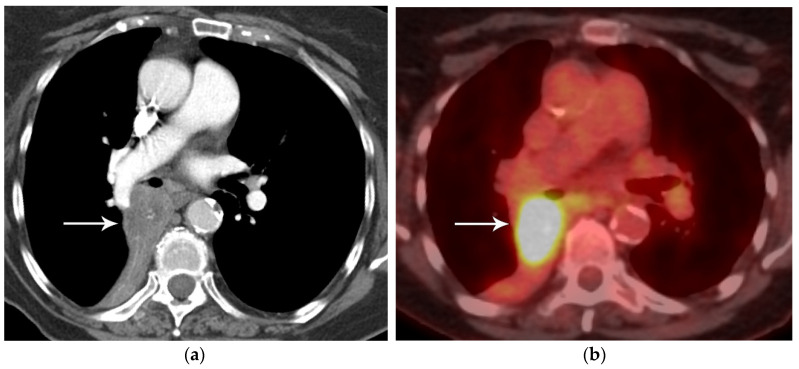
T2 disease. (**a**) Contrast-enhanced CT shows right lower lobe collapse due to central obstructing tumor (arrow). (**b**) Axial PET/CT shows FDG avidity of the primary tumor, differentiating it from adjacent atelectatic lung. Tumor size of 3.5 cm is T2a; lobar/lung atelectasis is also T2 disease. As there is no nodal and no distant metastasis, the final stage is stage IB.

**Figure 2:**
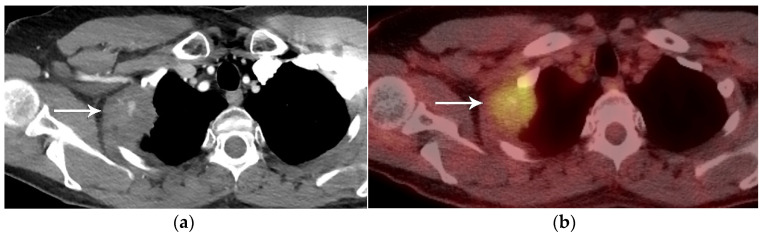
T3 disease. (**a**) Contrast-enhanced CT, and (**b**) axial PET/CT show the FDG-avid right upper lobe 4 cm primary tumor invading the chest wall (arrow). The size of 4 cm is T2a disease while chest wall invasion is T3 disease. With no nodal or distant metastases, the stage is IIB.

**Figure 3:**
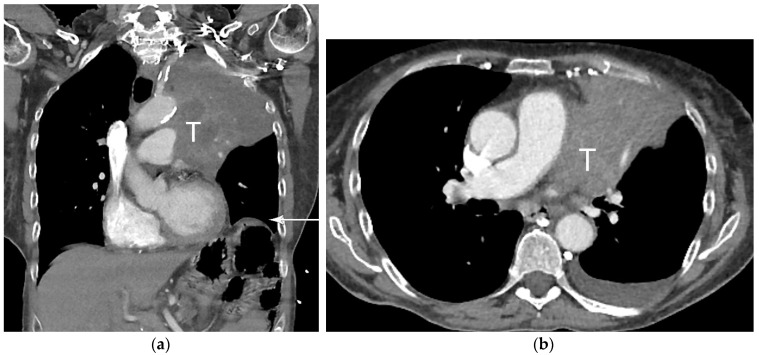
T4 disease. (**a**) Axial contrast-enhanced CT, and (**b**) coronal CT show 10 cm left upper lobe primary tumor (T) invading the mediastinum. Involvement of the left phrenic nerve resulted in elevation of the left hemidiaphragm (arrow). Involvement of the phrenic nerve is T3 disease. However, both size of greater than 7 cm and mediastinal invasion are T4 descriptors, dictating the T classification in this case.

**Figure 4:**
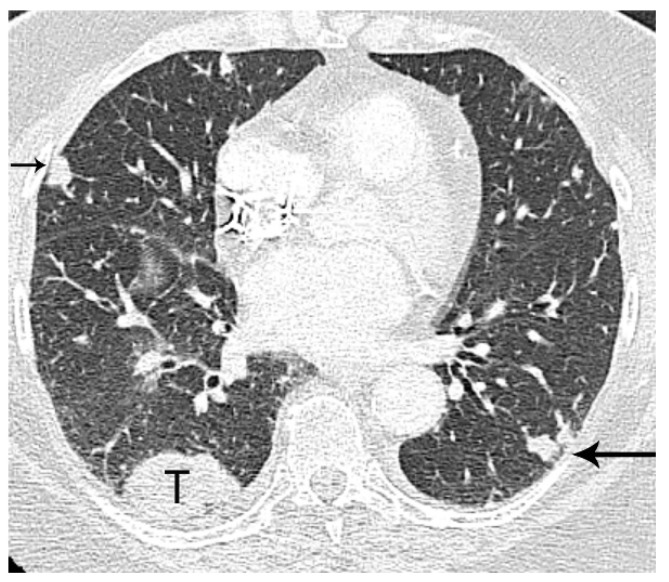
M1a disease, intrathoracic metastasis. Contrast-enhanced CT shows a 4 cm primary tumor (T) in the right lower lobe. Separate nodule in the right middle lobe (short arrow) as the primary tumor is T4 disease. Separate nodule (long arrow) in the left lower lobe (contralateral lung) as the primary tumor is M1a disease.

In addition to size, tumor location and the degree of local invasion are assessed in TNM staging and have the potential to increase the T classification. T1 tumors show no invasion into the lobar or more proximal bronchi. T2 tumors include lesions that show evidence of invasion of a main bronchus regardless of the distance from the carina [3]. Additionally, lesions with invasion of the visceral pleura, partial or complete lung atelectasis, or pneumonitis, are classified as T2 [3] (Figure 1). T3 tumors include lesions that show direct invasion of the parietal pleura, chest wall, phrenic nerve, or parietal pericardium (Figure 2). Lesions of any size that invade the mediastinum, diaphragm, heart, great vessels, recurrent laryngeal nerve, vertebrae, or carina are classified as T4 [3] (Figure 3). For patients with separate tumor nodules, location is important. A separate lung nodule(s) in the same lobe as the primary tumor is considered T3. A separate lung nodule(s) in the same lung but different lobe from the primary tumor is considered T4 [3]. A separate nodule(s) in the contralateral lung to the primary tumor is considered intrathoracic metastatic disease M1a (Figure 4).

FDG PET/CT, a combination of anatomical and functional imaging, is widely used in the staging of lung cancer. One benefit is the detection of recurrent laryngeal nerve involvement (T4), which manifests as ipsilateral vocal cord paralysis. In patients with recurrent laryngeal nerve involvement who are talking either before or during the PET/CT scan, there is FDG uptake in the normal vocal cord and lack of FDG uptake in the paralyzed cord. Another benefit of the functional data provided by FDG PET/CT is the ability to distinguish central obstructing primary tumors from adjacent atelectasis and post-obstructive consolidation, an imaging feature that is helpful to target the tumor for radiation therapy planning [16] (Figure 1).

**Figure 5:**
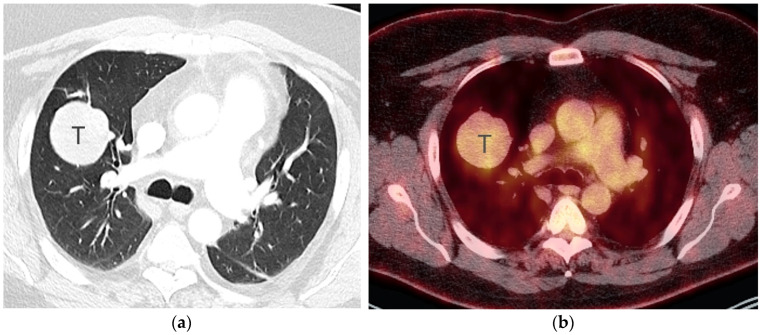
Bronchopulmonary carcinoid. (**a**) CT shows a 5 cm well-circumscribed solid mass (T) in the right middle lobe. (**b**) Axial PET/CT shows the right middle lobe lesion (T) has low-grade FDG uptake, similar to that of the mediastinum. Biopsy showed low-grade neuroendocrine tumor.

**Figure 6:**
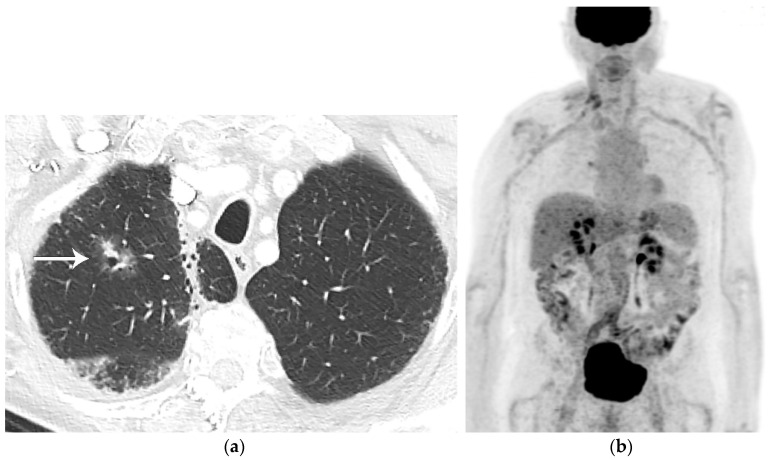
Lung adenocarcinoma. (**a**) CT shows a right upper lobe 2 cm part-solid nodule with focal “bubbly” internal lucencies and a solid component along the anterolateral aspect. Radiation fibrosis is noted in the right apex medially. (**b**) Whole-body PET shows the nodule is not FDG-avid. Biopsy revealed well-differentiated adenocarcinoma. Part-solid lung adenocarcinomas may not be FDG avid due to slow cell proliferation or poor cellularity.

FDG PET/CT is subject to both false negative and false positive results. False negative results can be observed with carcinoid tumors and some cases of early-stage disease (pre-invasive and minimally invasive lung adenocarcinoma) [17] (Figure 5 and Figure 6). In a study of 550 patients with stage I lung adenocarcinoma, using maximal standardized uptake value (SUVmax) cutoff of 2.5 to discriminate a positive from a negative result on FGD PET/CT, 17.6% of patients had a false negative FDG PET result where the primary tumor had SUVmax of less than 2.5 [17]. Lung adenocarcinoma with a lepidic pattern on pathology have a tendency toward false negative FDG PET findings [17]. There is evidence that focal FDG uptake correlates with tumor invasion in both pure ground-glass and part-solid malignancies, although many pre-invasive and minimally invasive adenocarcinomas may be below the resolution of PET due to their small size [18,19]. False positive FDG PET results can be seen with nonneoplastic processes such as infections, hamartomas, granulomatous disease, sarcoidosis, amyloidosis, lung parenchymal or pleural fibrosis, and round atelectasis [20].

## 3. N Classification

**Figure 7:**
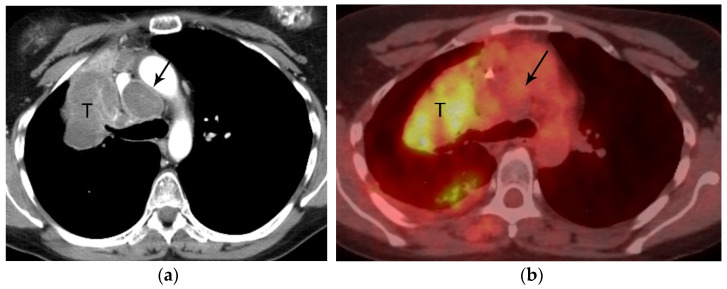
N2 nodal metastasis. (**a**) CT shows a 6.5 cm tumor (T) in the right upper lobe and low attenuation right paratracheal adenopathy (arrow). (**b**) PET/CT shows FDG avidity of the primary tumor (T) but the biopsy-proven N2 ipsilateral nodal metastasis is not FDG avid. Necrotic nodal metastases can give false negative results on PET/CT.

**Figure 8:**
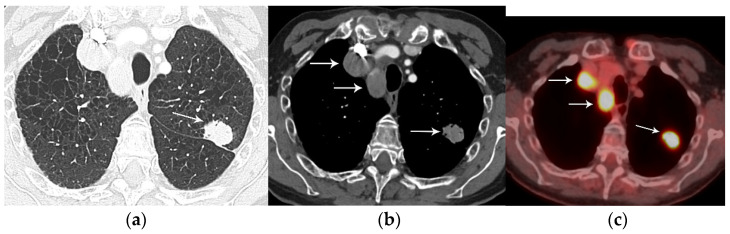
N3 nodal metastases. (**a**) CT shows a 2.5 cm tumor (arrow) in the left upper lobe. (**b**) Contrast-enhanced CT and (**c**) axial fused PET/CT show FDG-avid adenopathy in the right mediastinum in the paratracheal and prevascular regions (horizontal arrows). Biopsy confirmed nodal metastases in N3 (right paratracheal). Staging is T1N3M0, stage IIIB.

**Figure 9:**
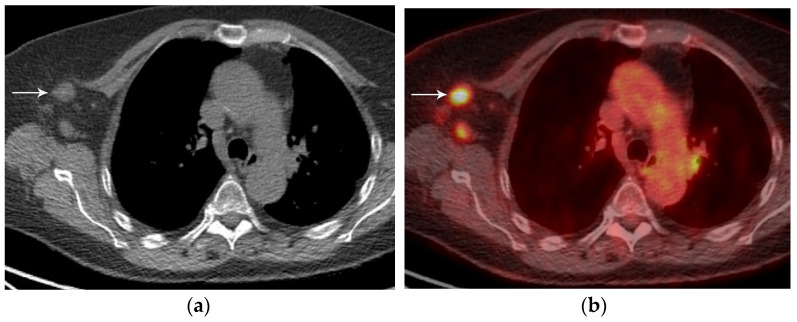
Left lung cancer and right axillary nodal metastases. (**a**) CT, and (**b**) axial PET/CT show FDG-avid right axillary adenopathy (arrow). Biopsy confirmed metastatic disease from the left lung cancer. The N status represents regional spread of disease. Lymph nodes not addressed in N classification, such as internal mammary, axillary, and retroperitoneal, represent distant metastatic disease.

Nodal status describes the regional spread of disease to intrathoracic lymph nodes and is a reliable indicator of prognosis [21]. Currently, nodal classification depends on the anatomic location of metastatic lymph nodes, and clinical staging is based on information from CT, PET/CT, endoscopic ultrasound (EUS), endobronchial ultrasound (EBUS), and mediastinoscopy [21]. Nodal classification is as follows: N0 (no involvement of regional lymph nodes), N1 (involvement of ipsilateral peribronchial, interlobar, or hilar lymph nodes), N2 (involvement of ipsilateral mediastinal lymph nodes), and N3 (contralateral mediastinal, contralateral hilar, or supraclavicular nodal involvement) [21] (Figure 7 and Figure 8). Patients with N0 or N1 disease can be referred to surgery while those with N2 or N3 disease require multimodality treatment. Other lymph nodes, not addressed in the current nodal staging system, are considered distant (M) metastatic disease (Figure 9). These lymph nodes include cervical, axillary, internal mammary, diaphragmatic, and retroperitoneal lymph nodes.

The TNM staging system is used around the world in a variety of practice environments, and as such, does not stipulate the use of advanced imaging modalities for clinical staging. As the availability and accessibility of imaging modalities vary in different geographic locations, awareness of recommendations and an understanding of the strengths and limitations of these imaging modalities are crucial to optimize clinical nodal staging. The American Society of Clinical Oncology (ASCO) recommends a CT of the chest and upper abdomen (with intravenous contrast, unless contraindicated) be performed, and if no distant metastases are detected, this should be followed by FDG PET/CT [22]. The National Comprehensive Cancer Network recommends FDG PET/CT for the initial evaluation of hilar and mediastinal lymph nodes, due to its superior sensitivity and specificity compared to CT [22].

**Figure 10:**
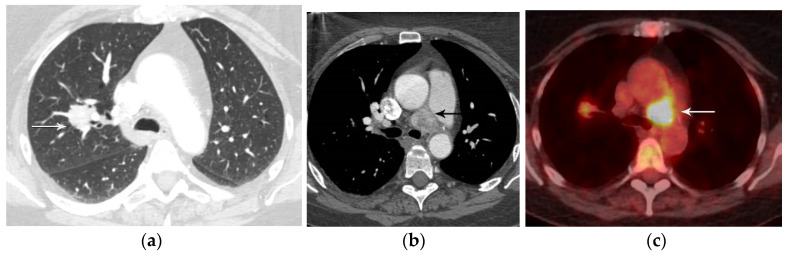
Paraganglioma mimicking N3 nodal metastasis. (**a**) CT shows a right upper lobe primary lung cancer (arrow). (**b**) Contrast-enhanced CT shows enhancing soft tissue in the left lower paratracheal region (arrow). (**c**) PET/CT shows the left mediastinal soft tissue is FDG avid, suspicious for N3 contralateral mediastinal nodal metastasis. Biopsy of the left paratracheal mass showed paraganglioma.

Malignant lymph nodes are identified on CT based on size with short axis diameter of greater than 1 cm and on PET/CT based on SUVmax of 2.5 or greater or based on visual assessment of SUV greater than mediastinal background [22]. PET is more accurate than CT for nodal staging with sensitivity of 0.85 and specificity of 0.90 compared to pooled sensitivity of CT of 0.61 and specificity of 0.79 [22]. The use of dual time point imaging is helpful and has been proven to be a good discriminator between malignant and benign lymph nodes. Nevertheless, false positive PET results due to nonneoplastic infectious or inflammatory processes can lead to misinterpretation and inaccurate staging. Another pitfall relates to nodal metastases that are not FDG avid due to central necrosis (Figure 7). According to the ASCO guidelines, the inaccuracy of imaging alone, either CT or FDG PET/CT, in nodal staging dictates that when curative intent treatment is planned, lymph nodes suspicious for metastases, even FDG-avid lymph nodes, must be confirmed pathologically whenever feasible (Figure 10). This is particularly important when the pretest probability of N2 nodal involvement is intermediate [22]. Thus, imaging plays an important role in selection of the biopsy site to verify the highest possible disease stage and to maximize tissue yield [22].

The TNM-8 nodal staging system is based solely on the location of involved lymph nodes and did not address tumor burden or number of lymph nodes involved. Recent studies support the use of the number and location of lymph node stations or lymph node zones as new parameters to categorize the extent of nodal involvement to improve prognostication [21].

In a study involving 3971 patients that subdivided the N classification into categories that included single-station N2 and multiple-station N2, a multivariate analysis revealed that pathologic lymph node stations or zones independently predicted overall survival and freedom from recurrence [23]. In another study, the number of involved lymph nodes and lymph node zones were also found to be useful prognostic discriminators [24].

Magnetic resonance imaging (MRI) short tau inversion recovery (STIR) turbo spin-echo (SE) sequences as well as diffusion-weighted imaging (DWI) have shown potential to help guide nodal staging [25]. For STIR imaging, the inversion time is approximately 80–150 ms. At these T1 values, longitudinal magnetization for virtually all tissues is negative when a 90° radiofrequency pulse is applied. Recovery begins promptly for most tissues. After the second 90° pulse, the T1 value of the tissue increases together with the tissue’s relative signal intensity, as does the T2 value. The T1 contrast and the T2 contrast are additive. Studies have shown significant differences between malignant and benign nodes in terms of their T1 and T2 relaxation times. Because many pathologic lesions show an increase in both T1 and T2, the addition of these two types of contrast with the STIR sequence produces a higher net tissue contrast. STIR imaging with a shorter time to echo and shorter echo train length than T1 weighted imaging has shown promise in nodal staging. Diffusion-weighted imaging can be useful for the quantitative and qualitative evaluation of nodal metastasis in lung cancer patients. Sensitivity, specificity, and accuracy of DWI ranged from 69.2% to 100%, 88% to 100%, and 71% to 94%, respectively [25].

Another potential area of study pertains to artificial neural networks. Experienced readers of PET/CT use interpretive skills and imaging features of lymph nodes, namely their location, FDG uptake, size, and relation to the primary lung tumor, to differentiate benign from malignant lymph nodes when staging lung cancer. Artificial neural networks have been used to emulate the accuracy of experienced readers. Machine learning models are in development to differentiate the clinically relevant categories N0/N1 and N2/N3 using variables from FDG PET/CT images and clinical and pathological data that can be obtained in any patient with routinely available tools. The features include lymph node SUVmax, lymph node short axis diameter, primary tumor diameter, and patient age [26].

## 4. M Classification

**Figure 11:**
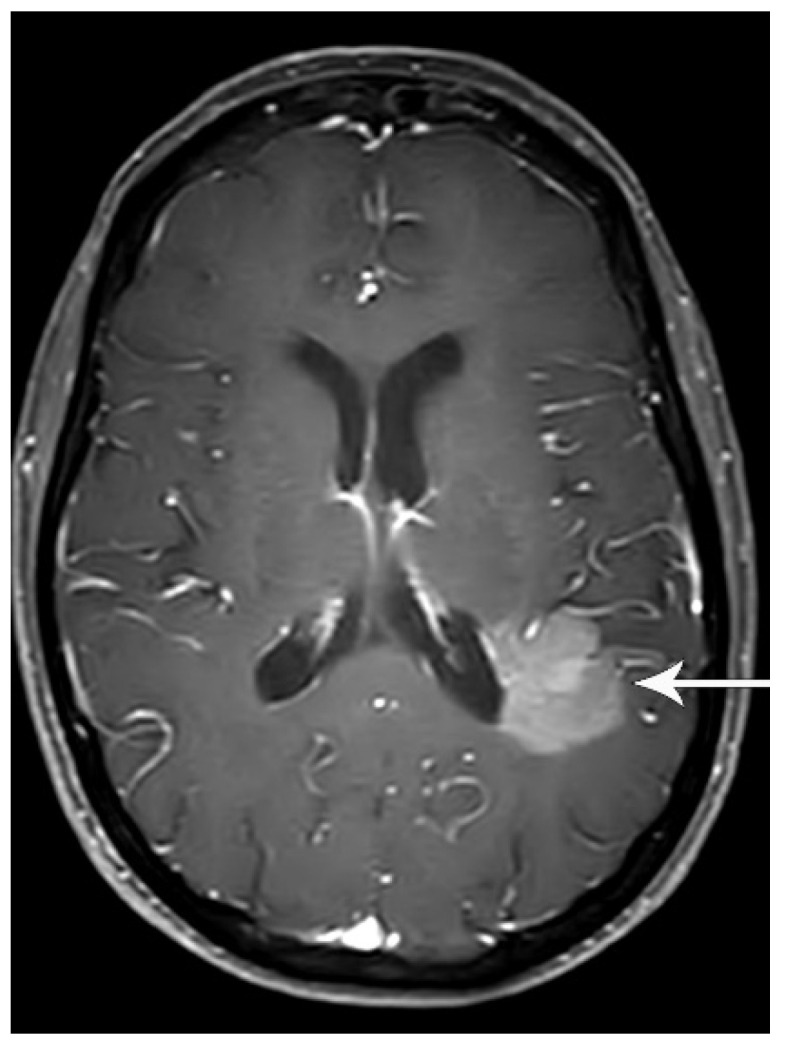
M1b disease, single extrathoracic metastasis. Contrast-enhanced brain MRI shows a left parietal enhancing metastasis (arrow). A single focus of extrathoracic metastasis is M1b disease, which constitutes stage IVA.

**Figure 12:**
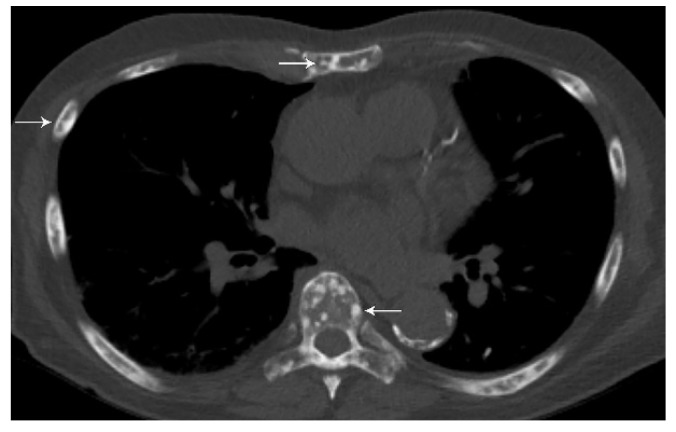
M1c disease, multiple extrathoracic metastases. CT shows multiple sclerotic bone metastases in the ribs, sternum, and spine (arrows). M1c disease, multiple extrathoracic metastases, constitutes stage IVB.

Distant metastasis is seen in 21% of patients with newly diagnosed NSCLC [27]. The M classification describes whether there is metastatic disease to intra- and extrathoracic organs. M0 indicates that there is no distant metastasis. M1a signifies intrathoracic metastases and includes pleural/pericardial effusion or nodule(s), contralateral tumor nodule(s), or a combination of these findings [28] (Figure 4). In TNM-8, the classification of M1b was revised as a single extrathoracic metastatic site, such as a single metastatic lesion in the brain, liver, bone, peritoneum, skin, adrenal gland, or a distant lymph node [28] (Figure 11). M1c denotes multiple distant metastatic lesions in a single organ or two or more lesions in multiple organs [28] (Figure 12). The separation of distant metastases into two categories (M1b and M1c) was based on the prognostic differences gleaned from a prospective data set with definitions for an oligometastatic disease subset [3].

The advantage of using PET/CT in lung cancer preoperative staging is the detection of occult distant metastatic disease (with common sites including the liver, adrenal glands, and bones), sparing the patient from futile aggressive local therapy [29,30]. A meta-analysis of four studies involving 360 patients showed a pooled sensitivity of 0.77 and specificity of 0.95 in the detection of extrathoracic metastatic disease [31]. In one study, 17% of patients with clinical stage III lung cancer had unexpected stage IV disease detected by FDG PET/CT [32].

FDG PET is useful for the detection and differentiation of adrenal lesions. Metser et al. reported that FDG PET can differentiate metastasis and benign adenomas using an SUV cutoff of 3.1 with sensitivity, specificity, positive predictive value (PPV), and negative predictive value (NPV) of 98.5%, 92%, 89.3%, and 98.9%, respectively [33]. When using a threshold of ≤10 Hounsfield units to identify benign adrenal adenomas on the CT component of FDG PET/CT, the sensitivity, specificity, PPV, and NPV were increased to 100%, 98%, 97%, and 100%, respectively [33]. In a retrospective study of 38 patients, Blake et al. reported that all malignant adrenal lesions demonstrated FDG activity greater than that of the liver, with a mean adrenal to liver ratio of 4.04 (range 1.53–17.08) [34]. Finally, in a meta-analysis of 21 studies including 1217 patients, Boland et al. showed that PET/CT was highly sensitive (mean 0.97) and specific (mean 0.91) for differentiating malignant from benign adrenal lesions [35].

In the detection of bone metastasis in patients with NSCLC, FDG PET/CT, and FDG PET are superior to MRI and bone scintigraphy. A meta-analysis of seventeen studies (of which nine studies used FDG PET/CT, nine studies used FDG PET, six studies used MRI, and sixteen studies used bone scintigraphy) assessed 2940 patients with lung cancer. The meta-analysis reported the pooled sensitivity for the detection of bone metastasis using FDG PET/CT, FDG PET, MRI, and bone scintigraphy were 0.92, 0.87, 0.77, and 0.86, respectively [36]. The pooled specificity for the detection of bone metastasis from lung cancer using FDG PET/CT, FDG PET, MRI, and bone scintigraphy were 0.98, 0.94, 0.92, and 0.88, respectively [36]. Bone is the third most common site of metastatic disease after liver and adrenal [37]. On CT, lung cancer bone metastases have variable lytic, sclerotic or mixed appearance. MRI allows the detection of smaller lesions, bone marrow infiltration, and epidural invasion with potential for neurologic compromise [38]. Whole-body MRI using diffusion weighted imaging (DWI) is emerging as a reliable technique to screen patients for metastatic bone disease [39]. One study reported that the whole-body MRI staging pathway had similar accuracy to standard staging pathways, improved staging efficiency, and had lower staging costs [40].

In the detection of central nervous system (CNS) metastasis, FDG PET/CT is limited due to the physiologic uptake of glucose in the brain. Studies have shown that 1.6–21% of asymptomatic patients with stage III lung cancer have clinically occult CNS metastases [41,42,43]. Accordingly, the American Society of Clinical Oncology recommends dedicated brain imaging preferably with contrast-enhanced brain MRI (contrast-enhanced head CT scan may be used if MRI is contraindicated) in patients with clinical stage III lung cancer [22].

Hepatic metastases are reported in 3.8% of newly diagnosed NSCLC patients with a median overall survival of approximately 4 months [44]. CT, MRI, and PET are typically used for the diagnosis of liver metastases. On CT, liver metastases can show a variety of imaging appearances with the administration of intravenous contrast and can mimic benign lesions such as hemangiomas or adenomas. MRI adds value due to its higher accuracy in the detection of liver metastasis compared to CT [45].

**Figure 13:**
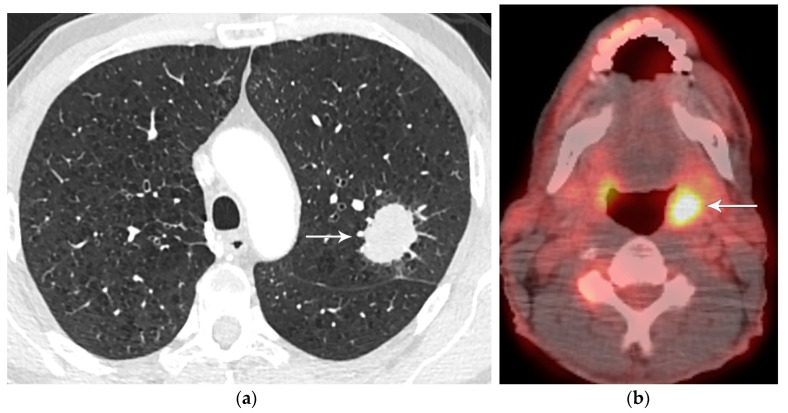
Second malignancy. (**a**) Contrast-enhanced CT shows a left upper lobe lung malignancy (arrow). Biopsy showed adenocarcinoma. (**b**) Axial PET/CT shows FDG-avid focus in the left tonsil (arrow). Biopsy revealed squamous cell cancer. With no nodal and distant metastases, the patient proceeded to left upper lobectomy. FDG-avid lesions suspicious for metastases in NSCLC patients being considered for surgical resection should be biopsied to obtain a histopathologic diagnosis. PET/CT is useful in the detection of extrathoracic metastases as well as second primaries.

**Figure 14:**
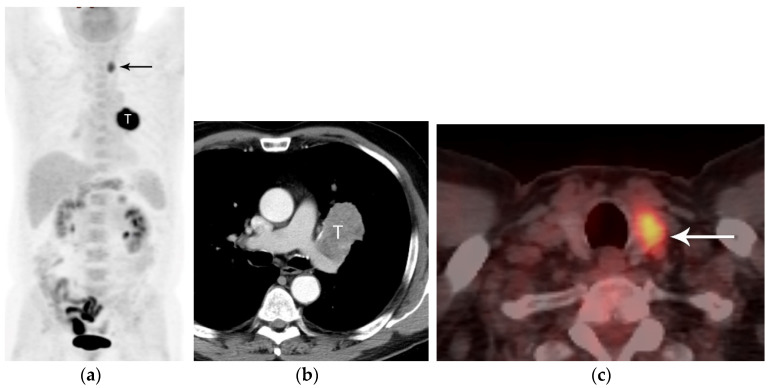
FDG-avid lesion unrelated to lung cancer. (**a**) Whole-body PET shows the left upper lobe primary tumor (T) and an FDG-avid focus in the left neck (arrow). (**b**) CT shows the left upper lobe tumor (T). (**c**) Axial PET/CT shows the FDG-avid focus is localized to the left lobe of the thyroid. Biopsy showed colloid nodule.

In the interpretation of FDG PET/CT in lung cancer staging, it is important to be aware of a potential pitfall regarding FDG-avid lesions unrelated to lung cancer. These findings include FDG-avid benign lesions or second malignancies that can be misinterpreted as distant metastases (Figure 13 and Figure 14). One study reported that 9% of solitary FDG-avid extrathoracic lesions identified on staging PET for lung cancer were benign, and 37% were unrelated to the lung cancer [46]. For presumed stage III (T4,N0 or T3,N1-3 or T1-4,N2-3) NSCLC patients, any suspected metastatic site detected on CT or PET/CT should be verified pathologically with a biopsy, as per ASCO recommendations. Typically, biopsy sites should be chosen to corroborate the highest possible disease stage and to maximize tissue yield [22].

In terms of future directions, gallium 68-labeled fibroblast-activation protein inhibitor (FAPI) has recently been introduced as a promising tumor imaging agent. In a recent study of 34 patients, FAPI PET/CT outperformed FDG PET/CT in staging lung cancer, particularly in the detection of metastases to the brain (23 vs. 10), lymph nodes (356 vs. 320), bone (109 vs.91), and pleura (66 vs. 35) [47].

## 5. Resectability

In the assessment of the primary tumor, the presence and degree of surrounding tissue invasion, as well as involvement of the central airways and great vessels, is important for surgical planning. For example, involvement of the origin of the lobar bronchus or main bronchus may necessitate sleeve resection or pneumonectomy [48]. In lung cancer staging, it is essential to differentiate resectable from unresectable tumors as the latter is treated with chemotherapy, immunotherapy, and/or radiation therapy [49]. Unresectable disease includes select T4 lesions, N3 disease, and any distant metastasis. Involvement of the main pulmonary artery, a T4 descriptor that can be resected in some centers, may necessitate pneumonectomy rather than lobectomy in order to obtain clear surgical margins [50]. According to the ASCO guidelines, for selected patients with T4N0 disease (by size or extension), surgical resection may be offered if medically and surgically feasible following multidisciplinary review [22]. N1 disease (ipsilateral peribronchial or hilar nodes) is usually resectable. Ipsilateral non-bulky, single station mediastinal or subcarinal adenopathy (N2) may be resectable (usually after induction chemotherapy or chemoradiation). Contralateral mediastinal adenopathy and scalene or supraclavicular adenopathy (N3) and metastatic disease are unresectable.

## 6. PET/CT for Lung Cancer

The strength of FDG PET/CT in lung cancer pre-operative staging is the detection of occult extrathoracic metastases, sparing patients unnecessary surgery. In a randomized controlled trial, Fischer et al. found that the use of FDG PET/CT decreased the total number of thoracotomies and futile thoracotomies (defined as any of the following: benign lung lesion, stage IIIA or higher, inoperable T3 or T4 disease, or recurrent disease or death from any cause within 1 year after randomization) [51]. FDG PET/CT upstaged 13.8% of patients, compared with 6.8% of patients who were staged using CT and a whole-body bone scan, in a multicenter randomized controlled trial [52].

The United States Food and Drug Administration approved the use of gallium-68 tetraazacyclododecane tetraacetic acid (DOTA)–octreotate ([^68^ Ga] Ga-DOTA-TATE) for PET imaging of neuroendocrine tumors in 2016 [6]. Bronchopulmonary carcinoid tumors express somatostatin receptors (SSTR). [^68^ Ga] Ga-DOTA-TATE has shown higher and more selective uptake in carcinoid tumors. In contrast, carcinoid tumors typically show little to no FDG uptake [6]. Knowledge of the normal radiotracer biodistribution is important to avoid interpretation errors. For [^68^ Ga] Ga-DOTA-TATE and similar somatostatin receptor ligands, the spleen demonstrates the highest level of radiotracer activity, followed by the adrenal glands, pituitary, kidneys, and liver. In combination with FDG PET/CT, [^68^ Ga] Ga-DOTA-TATE PET/CT can noninvasively assess tumor heterogeneity, especially in intermediate grade carcinoids, for personalized management of patients [53].

One future area of study is FDG PET/CT radiomics, the practice of uncovering and analyzing the invisible data embedded in medical images. These quantitative parameters include features of the lesion and surrounding tissues pertaining to morphology, intensity, and texture. Radiomic features can then be correlated with clinical, histological, and molecular findings. Over the last decade, FDG PET/CT radiomics has been applied to improve staging accuracy, as well as to predict histology, tumor biomarkers, response to therapy, and prognosis [54,55,56,57,58,59]. Mu et al. reported the value of pretreatment FDG PET/CT radiomics in predicting severe immune-related adverse events among patients with advanced non-small cell lung cancer treated with immunotherapy, which is important in optimizing treatment strategies and mitigating future complications with early interventions [57]. As the number of the extracted features is too large to be assessed by traditional statistical analytical methods, artificial intelligence, and machine learning can be used to develop more accurate predictive models [60].

## 7. Conclusions

The TNM staging system is applicable to the staging of non-small cell lung cancer, small cell lung cancer, and bronchopulmonary carcinoid tumors. Imaging is integral in accurate clinical staging and is essential to guide treatment strategies to optimize patient outcomes. CT is widely available and used for lung cancer diagnosis and staging. FDG PET/CT can improve upon staging with CT alone, particularly for the detection of nodal and distant metastases. Limitations of FDG PET/CT include lung cancers that are not FDG avid including carcinoid tumors and indolent lung adenocarcinomas, and non-neoplastic infectious or inflammatory conditions that are FDG avid. Knowledge of the strengths and weaknesses of various imaging modalities, the nuances of the TNM staging classification, and potential pitfalls is important to avoid misinterpretation in lung cancer staging.

## Figures and Tables

**Table 1:** IASLC Lung Cancer Staging Project T, N, and M Descriptors for the TNM-8 Classification of Lung Cancer.

T—Primary Tumor		
Category	Subcategory	Descriptors
TX		Primary tumor cannot be assessed, or tumor is proven by the presence of malignant cells in sputum or bronchial washings but not visualized by imaging or bronchoscopy
T0		No evidence of primary tumor
Tis		Carcinoma in situ: Tis(AIS): adenocarcinoma Tis(SCIS): squamous cell carcinoma
T1		Tumor 3 cm or less in greatest dimension, surrounded by lung or visceral pleura, without bronchoscopic evidence of invasion more proximal than the lobar bronchus (i.e., not in the main bronchus). The uncommon superficial spreading tumor of any size with its invasive component limited to the bronchial wall, which may extend proximal to the main bronchus, is also classified as T1a.
	T1mi	Minimally invasive adenocarcinoma
	T1a	Tumor 1 cm or less in greatest dimension
	T1b	Tumor more than 1 cm but not more than 2 cm in greatest dimension
	T1c	Tumor more than 2 cm but not more than 3 cm in greatest dimension
T2		Tumor more than 3 cm but not more than 5 cm; or tumor with any of the following features. T2 tumors with these features are classified T2a if 4 cm or less, or if size cannot be determined; and T2b if greater than 4 cm but not larger than 5 cm. Involves main bronchus regardless of distance to the carina, but without involving the carina Invades visceral pleura Associated with atelectasis or obstructive pneumonitis that extends to the hilar region, either involving part of the lung or the entire lung
	T2a	Tumor more than 3 cm but not more than 4 cm in greatest dimension
	T2b	Tumor more than 4 cm but not more than 5 cm in greatest dimension
T3		Tumor more than 5 cm but not more than 7 cm in greatest dimension or one that directly invades any of the following: parietal pleura (PL3), chest wall (including superior sulcus tumors), phrenic nerve, parietal pericardium; or associated separate tumor nodule(s) in the same lobe as the primary
T4		Tumors more than 7 cm or one that invades any of the following: diaphragm, mediastinum, heart, great vessels, trachea, recurrent laryngeal nerve, esophagus, vertebral body, carina; separate tumor nodule(s) in a different ipsilateral lobe to that of the primary
**N—Regional Lymph Nodes**		
NX		Regional lymph nodes cannot be assessed
N0		No regional lymph node metastasis
N1		Metastasis in ipsilateral peribronchial and/or ipsilateral hilar lymph nodes and intrapulmonary nodes, including involvement by direct extension
N2		Metastasis in ipsilateral mediastinal and/or subcarinal lymph node(s)
N3		Metastasis in contralateral mediastinal, contralateral hilar, ipsilateral or contralateral scalene, or supraclavicular lymph node(s)
**M—Distant Metastasis**		
M0		No distant metastasis
M1		Distant metastasis
	M1a	Separate tumor nodule(s) in a contralateral lobe; tumor with pleural nodules or malignant pleural or pericardial effusion. Most pleural (pericardial) effusions with lung cancer are due to tumor. In a few patients, however, multiple microscopic examinations of pleural (pericardial) fluid are negative for tumor, and the fluid is non-bloody and is not an exudate. Where these elements and clinical judgment dictate that the effusion is not related to the tumor, the effusion should be excluded as a staging descriptor.
	M1b	Single extrathoracic metastasis in a single organ and involvement of a single distant (non-regional) node
	M1c	Multiple extrathoracic metastases in one or several organs

**Table 2:** Stage groupings for NSCLC.

T or M Stage		N0	N1	N2	N3
T1	T1a	IA1	IIB	IIIA	IIIB
	T1b	IA2	IIB	IIIA	IIIB
	T1c	IA3	IIB	IIIA	IIIB
T2	T2a	IB	IIB	IIIA	IIIB
	T2b	IIA	IIB	IIIA	IIIB
T3	T3	IIB	IIIA	IIIB	IIIC
T4	T4	IIIA	IIIA	IIIB	IIIC
M1	M1a	IVA	IVA	IVA	IVA
	M1b	IVA	IVA	IVA	IVA
	M1c	IVB	IVB	IVB	IVB

## Data Availability

Not applicable.
